# Transcriptome profiling of *Fagopyrum tataricum* leaves in response to lead stress

**DOI:** 10.1186/s12870-020-2265-1

**Published:** 2020-02-03

**Authors:** Lei Wang, Bei Zheng, Yong Yuan, Quanle Xu, Peng Chen

**Affiliations:** Department of Biochemistry & Molecular Biology, College of Life Sciences, Northwest A&F University, Yangling, 712100 Shaanxi China

**Keywords:** *Fagopyrum tataricum*, Lead stress, Transcriptome, Ultrastructural localization, Heterologous expression

## Abstract

**Background:**

Lead (Pb) pollution is a widespread environmental problem that is harmful to living organisms. Tartary buckwheat (*Fagopyrum tataricum*), a member of the family Polygonaceae, exhibits short growth cycles and abundant biomass production, could be an ideal plant for phytoremediation due to its high Pb tolerance. Here, we aimed to explore the molecular basis underlying the responses of this plant to Pb stress.

**Results:**

In our study, ultrastructural localization assays revealed that Pb ions primarily accumulate in leaf vacuoles. RNA deep sequencing (RNA-Seq) of tartary buckwheat leaves was performed on two Pb-treated samples, named Pb1 (2000 mg/kg Pb (NO_3_)_2_) and Pb2 (10,000 mg/kg Pb (NO_3_)_2_), and a control (CK). A total of 88,977 assembled unigenes with 125,203,555 bases were obtained. In total, 2400 up-regulated and 3413 down-regulated differentially expressed genes (DEGs) were identified between CK and Pb1, and 2948 up-regulated DEGs and 3834 down-regulated DEGs were generated between CK and Pb2, respectively. Gene Ontology (GO) and pathway enrichment analyses showed that these DEGs were primarily associated with ‘cell wall’, ‘binding’, ‘transport’, and ‘lipid and energy’ metabolism. The results of quantitative real-time PCR (qRT-PCR) analyses of 15 randomly selected candidate DEGs and 6 regulated genes were consistent with the results of the transcriptome analysis. Heterologous expression assays in the yeast strain *Δycf1* indicated that overexpressing CCCH-type zinc finger protein 14 (ZFP14) enhanced sensitivity to Pb^2+^, while 5 other genes, namely, metal transporter protein C2 (MTPC2), phytochelatin synthetase-like family protein (PCSL), vacuolar cation/proton exchanger 1a (VCE1a), natural resistance-associated macrophage protein 3 (Nramp3), and phytochelatin synthetase (PCS), enhanced the Pb tolerance of the mutant strain.

**Conclusion:**

Combining our findings with those of previous studies, we generated a schematic model that shows the metabolic processes of tartary buckwheat under Pb stress. This study provides important data for further genomic analyses of the biological and molecular mechanisms of Pb tolerance and accumulation in tartary buckwheat.

## Background

Lead (Pb) is one of the most toxic inorganic metal pollutants worldwide and is a persistent environmental contaminant. Pb is generated by many industrial processes and is subsequently discharged into soils, waters and the atmosphere, potentially inducing a broad range of toxic effects in living organisms [1–4]. As one of the non-essential ions, Pb toxicity in plants results in seed germination inhibition, restrained growth of seedings, wither, and crop yield reduction [5]. It penetrates plants through the roots and is transported to shoot tissues [6, 7], causing a number of toxic effects on plant morphology, including enzymatic reactions, chlorophyll biosynthesis, membrane permeability and a number of other metabolic processes [5, 8, 9]. Therefore, plants can serve as effective biological monitors and indicators for environmental quality assessments [10, 11]. Meanwhile, phytoremediation has been considered to be an inexpensive remediation technology to remove heavy metals from contaminated soils [12].

Currently, a number of plant species have been studied to understand the mechanism of Pb tolerance and phytoremediation, but differ with species in Pb uptake, transportation, accumulation and tolerance [13]. In this connection, Ferreyroa et al. [14] have characterized *Brassica napus* plant’s performance for metal accumulation and detoxification mechanism, and the results suggest a decrease in chlorophyll contents at low Pb concentration and cell damage at higher lead concentration. Additionally, *Acalypha indica* plant has been observed to show the physiological and biochemical changes under exposure to lead (100–500 mg L^− 1^) [15], but only grows in the tropics with a narrower adaptable. In a study, *Platanus acerifolia* has been demonstrated the molecular regulation mechanism of Pb accumulation and tolerance with 12 g L^− 1^ Pb (NO_3_)_2_, and considered to be well adapt to Pb pollution [16]. However, it is a perennial woody plant which needs to take years to purify the soil. Thus, the phytoremediation was restricted by the lack of Pb hyper-accumulators, relatively low biomass and poor adaptation ability.

Tartary buckwheat (*Fagopyrum tataricum*) belongs to the eudicot family Polygonaceae [17] that has been widely domesticated as a food and ornamental crop in some East Asian countries [18, 19]. Buckwheat is widely adaptable to low-fertility soils, exhibiting high biomass accumulation and short growth cycles [20]; additionally, it was showed that buckwheat accumulates more Pb in its shoots and especially in its leaves. Moreover, buckwheat accumulates lead without displaying symptoms of growth inhibition [21]. In a comparison of three different cultivars of buckwheat (Xiqiao No. 1, Jinqiao No. 1, and Jiujiang) treated with Pb^2+^, Jiujiang displayed few or no toxicity symptoms and exhibited fewer changes in relative cytoplasmic membrane permeability and chlorophyll content than the other assayed cultivars [22]. Tartary buckwheat has been reported to be rich in flavonoids and phenols [23], molecules that protect plants from the adverse effects of active free radicals resulting from Pb-induced oxidative stress [24]. Consequently, tartary buckwheat can be used as potential accumulator species for remediation of Pb contaminated environment. However, none of the genes and metabolic pathways involved in regulating the Pb stress response in tartary buckwheat have been identified, interfering our understanding of Pb tolerance mechanisms in this important accumulator crop.

Next-generation sequencing (NGS) techniques are become powerful tools for life science research and have promoted the rapid discovery of previously unknown genes. For example, RNA deep sequencing (RNA-Seq) has been used in molecular studies of the environmental stress responses of many plants. Wu et al. [25] reported the salt-responsive transcriptome of *Fagopyrum tataricum* and identified 455 DEGs involved in the salt stress response. It is an effective approach for data mining of a species whose genome database was not available. With respect to Pb toxicity, Tian et al. [26] analysed the transcriptome of *Louisiana iris* root and identified many important candidate genes and pathways in order to discover the mechanism related to the lead tolerance and accumulation. In maize, a number of transcription factor (TF) families that response to Pb exposure, including bZIP, ERF and GARP, were upregulate under Pb stress in the roots [27]. Moreover, the study demonstrated that several genes involved in the ABA biosynthetic pathway were upregulated in the roots and shoots of *Hirschfeldia incana* after treatment with Pb, suggesting that ABA-mediated signalling is potentially involved in the response of this plant to lead [28]. In addition, RNA-Seq analyses of the *Platanus acerifolia* have identified 16,246 DEGs associated with Pb exposure, including antioxidant enzymes, metal transporters and chelate proteins [16]. These results indicate that the regulatory network and defence system are complex and unique specificities in different plants under Pb stress.

Although tartary buckwheat has previously been characterized with respect to Pb tolerance and accumulation [22], as described above, the corresponding molecular mechanisms have not been deciphered. For example, the regulated gene responsible for Pb-induced protection has not been identified. When Pb is transported from soil to the vacuoles of leaves, the process involves multiple metabolic pathways; however, none of these pathways has been elucidated in buckwheat thus far. In the present study, we determined that the leaf is the main storage tissue by comparing the ultrastructural localization of Pb in different tissues. Then we used Illumina sequencing technology to gain a high-quality genome-wide transcript of tartary buckwheat leaves under Pb exposure and to identify crucial genes and pathways that are involved in tolerance mechanisms for this heavy metal, with the goal of applying this information to phytoremediation.

## Results

### Concentration and subcellular distribution of Pb in different tissues of tartary buckwheat

**Fig. 1:**
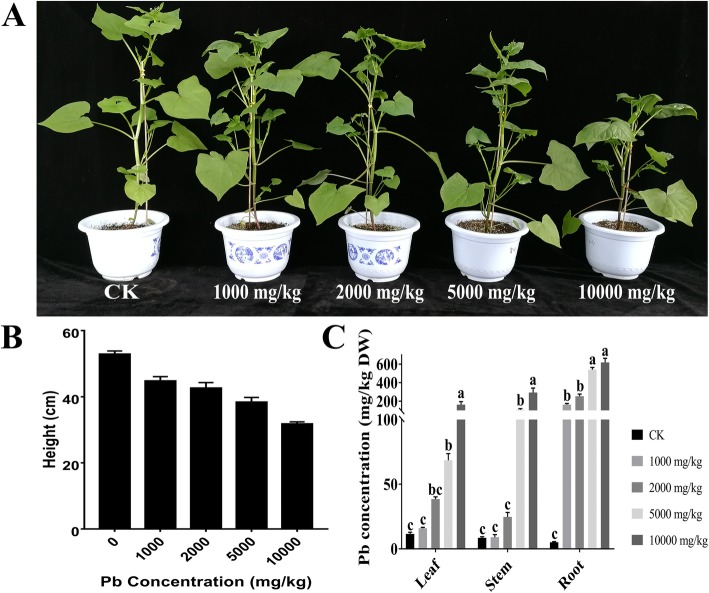
The response of *F. tataricum* to lead treatment. **a** The phenotype of *F. tataricum* growth on medium with different Pb concentrations. **b** The height of *F. tataricum* growth on medium with different Pb concentration. Plants were treated with 0, 1000, 2000, 5000, or 10,000 mg/kg Pb (NO_3_)_2_ for 30 days. **c** The Pb contents in the leaves, stems and roots of *F. tataricum* were determined by atomic absorption spectrophotometry (AAS). The data are presented as the mean ± SE (*n* = 3). Bars with different lowercase letters are significantly different at *P* < 0.05 (Tukey’s test)

Lead exposure up to 10,000 mg/kg reduced height but had no other effect on the buckwheat plants (Fig. 1a, b); this was the highest concentration assayed in subsequent experiments. Furthermore, lead exposure caused no change in MDA content, GSH content, soluble protein and SOD activity indicating the treatments did not cause damage to the leaf physiology (Additional file 1: Figure S1). Subsequently, the Pb content in different tissues was analysed (Fig. 1c), and the Pb content in the roots progressively increased when the Pb concentration exceeded 5000 mg/kg soil. In contrast, the Pb content in the leaves increased progressively at Pb concentrations ranging from 1000 to 10,000 mg/kg soil.

**Fig. 2:**
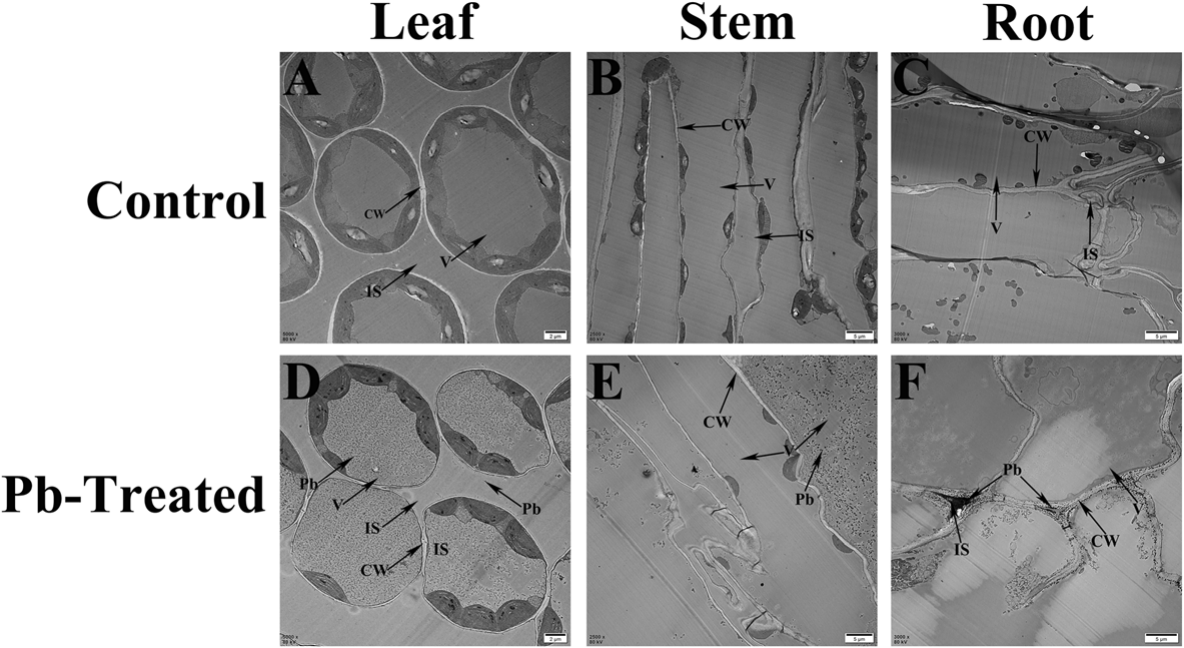
Ultrastructure of *F. tataricum* and Pb localization under the 10,000 mg/kg Pb treatment. A control leaf (**a**), stem (**b**) and root (**c**) compared to a Pb-treated leaf (**d**), stem (**e**) and root (**f**). IS, intercellular space; V, vacuole; CW, cell wall. (A, D, Bar = 2 μm; B, C, E, F, Bar = 5 μm)

The results of transmission electron microscopy (TEM) analyses revealed that Pb ions were primarily distributed within the vacuoles and walls of leaf cells, with fewer ions observed in the intercellular spaces (Fig. 2d). In parts of stem cells, some Pb ions were observed in vacuoles (Fig. 2e), whereas in the roots, Pb ions were primarily deposited in the cell walls and intercellular spaces (Fig. 2f). Based on these results, we chose to use leaves in subsequent experiments.

### De novo assembly and annotation

To identify genes associated with Pb tolerance and accumulation in the leaves of tartary buckwheat, three biological replicates each were selected from the non-treated, 2000 mg/kg Pb (NO_3_)_2_-treated and 10,000 mg/kg Pb (NO_3_)_2_-treated groups for cDNA library construction and transcriptome sequencing by using an Illumina HiSeq 4000 instrument.

An overview of the RNA-Seq reads derived from the sequencing results is presented in Table 1. For the three group samples, the average Q20 and GC content values of these clean reads were greater than 96 and 47%, respectively. A total of 88,977 unigenes with 125,203,555 unigene bases, a length range of 201–19,818 bp and an average length of 982.34 bp were assembled using the unigene database (Table 1; Additional file 2: Figure S2). A significant match in at least one of the above databases was obtained for 39,321 (44.18%) unigenes (Table 2). Among these unigenes, 27,071 (69%) unigenes were annotated in function. Regarding the alignment distribution, 18,434 (48.38%) of the annotated unigenes matched 14 species, and 33,080 (84.14%) unigenes matched a database sequence with a similarity of 60 to 100% (Additional file 3: Table S1).

**Table 1:** Throughput and quality of strand-specific RNA-seq of *F. tataricum* libraries

	CK	Pb1	Pb2
HiSeq Statistics			
Raw reads	48,264,504	51,792,400	47,292,888
Raw base (bp)	7,287,940,104	7,820,652,400	7,141,226,088
> Q20 of raw data (%)	97.07	97.29	96.84
Clean reads	47,737,814	51,208,144	46,731,827
Clean base (bp)	7,126,704,866	7,613,422,269	6,959,839,129
> Q20 clean reads (%)	97.68	97.91	97.50
GC percentage (%)	50.48	53.15	49.01
Assembly data (all the clean reads of the 6 libraries were assembled together)			
Total unigene number	88,977		
Total unigene base	125,203,555		
Percent GC	41.27		
Length of the largest unigene (bp)	19,818		
Length of the smallest unigene (bp)	201		
Average length	982.34		
N50	1730		
E90N50	1942

**Table 2:** Statistics for functional annotations

Database	Total unigenes	Annotated unigenes	Percentage
Pfam	88,977	34,945	39.27%
KEGG	88,977	26,443	29.72%
Swiss-Prot	88,977	37,824	42.51%
COG	88,977	8727	9.81%
GO	88,977	27,071	30.42%

### DEGs under Pb stress

**Fig. 3:**
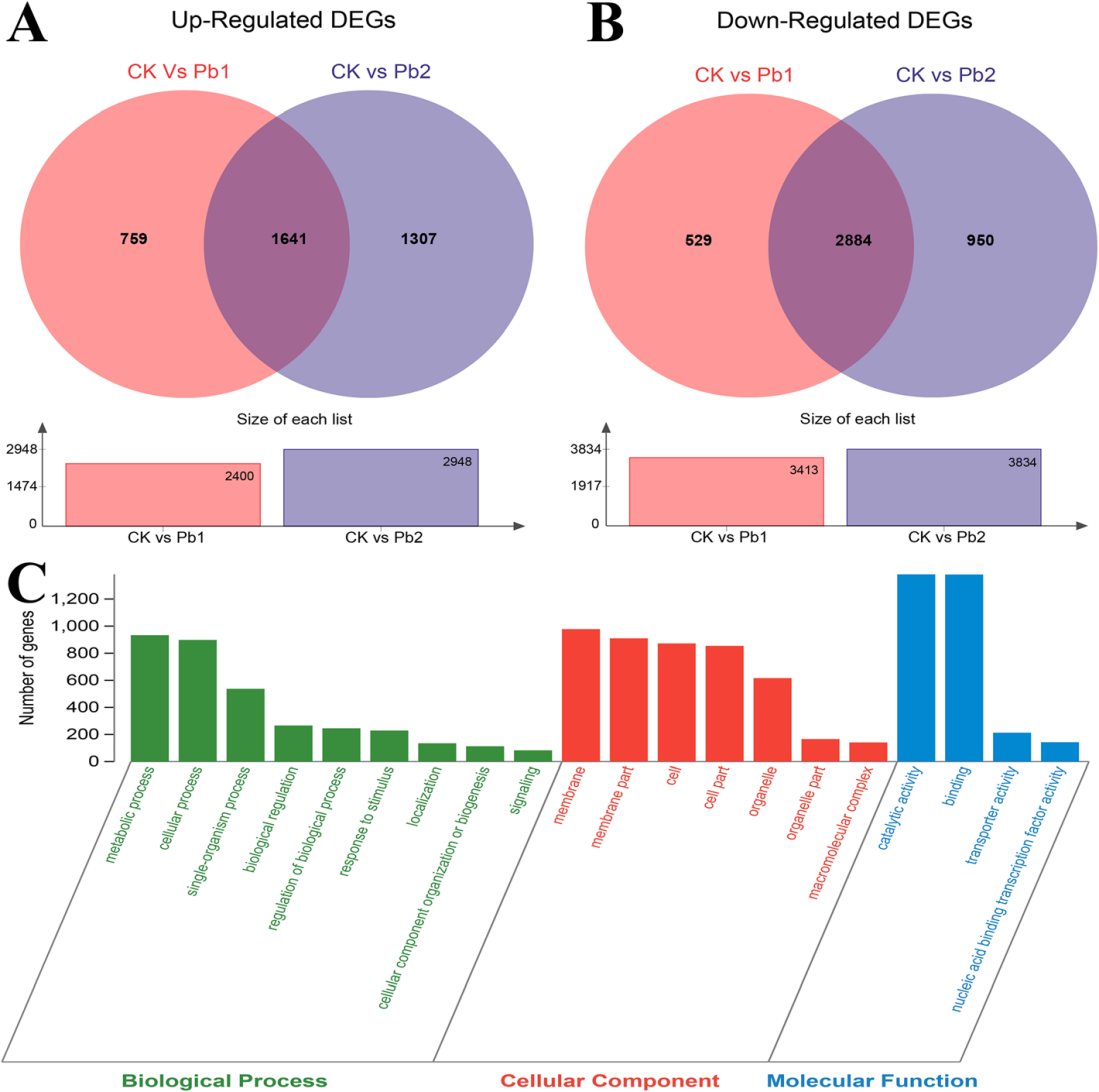
Venn diagram of differentially expressed genes (DEGs) for CK vs Pb1 and CK vs Pb2 and Gene Ontology classification of DEGs. **a**) Upregulated DEGs for CK vs Pb1 and CK vs Pb2; (**b**) Downregulated DEGs for CK vs Pb1 and CK vs Pb2; (**c**) GO classification of DEGs for CK vs Pb1 and CK vs Pb2. The x-axis represents GO terms belonging to three categories; the y-axis represents the gene numbers for each term

To identify unigenes that were induced by Pb stress, the three libraries were divided into two groups (CK vs Pb1 and CK vs Pb2), and 4525 common DEGs (Additional file 4: Figure S3) were identified in these comparisons. In total, we identified 1641 upregulated (Fig. 3a) and 2884 downregulated (Fig. 3b) unigenes under Pb stress. Among these 4525 DEGs, 3975 (87.84%) were annotated by the NR database.

A hypergeometric distribution was used to classify the DEGs into 20 level-2 GO terms (Fig. 3c). In detail, GO enrichment analysis identified 145 molecular function (MF) terms, 244 biological process (BP) terms and 64 cellular component (CC) terms (Additional file 5: Table S2). For these GO terms, significantly overrepresented (*P* < 0.05) terms in the MF category included catalytic ‘activity’ (GO:0003824), ‘binding’ (GO:0005488), ‘transporter activity’ (GO:0005215), and ‘nucleic acid binding transcription factor activity’ (GO:0001071). For BP terms, the most significantly overrepresented terms were ‘metabolic process’ (GO:0008152), ‘cellular process’ (GO:0009987), ‘single-organism process’ (GO:0044699) and ‘biological regulation’ (GO:0065007). ‘Membrane’ (GO:0016020), ‘membrane part’ (GO:0044425), ‘cell’ (GO:0005623), ‘cell part’ (GO:0044464) and ‘organelle’ (GO:0043226) were significantly overrepresented terms in the CC category. In summary, the identified unigenes in tartary buckwheat under Pb stress were primarily associated with catalytic activity, binding, metabolic processes and membrane components.

KEGG pathway enrichment analysis of CK vs Pb1 and CK vs Pb2 identified 106 enriched pathways (Additional file 6: Table S3), 17 of which were significantly enriched at *P* < 0.05 under Pb stress (Table 3). The four most common pathways responding to Pb stress were ‘plant hormone signal transduction’ (ko04075, 82), ‘plant-pathogen interaction’ (ko04626, 56), ‘MAPK signalling pathway - plant’ (ko04016, 65) and ‘phenylpropanoid biosynthesis’ (ko00940, 45). These results indicate that Pb stress in *F. tataricum* primarily influences pathways associated with energy metabolism, lipid metabolism, secondary metabolites, nonenzymatic antioxidants and oxidative phosphorylation.

**Table 3:** Common KEGG pathway enrichment for CK vs Pb1 and CK vs Pb2

Pathway	Pathway ID	DEGs	All Genes	*P*-value*
Plant hormone signal transduction	ko04075	82/1674	412/26443	0
Plant-pathogen interaction	ko04626	56/1674	305/26443	0
MAPK signalling pathway - plant	ko04016	65/1674	297/26443	0
Phenylpropanoid biosynthesis	ko00940	45/1674	243/26443	2.86336E-10
Carotenoid biosynthesis	ko00906	15/1674	53/26443	1.44839E-05
Starch and sucrose metabolism	ko00500	47/1674	360/26443	3.96627E-05
Sesquiterpenoid and triterpenoid biosynthesis	ko00909	11/1674	37/26443	0.000193512
Sulfur metabolism	ko00920	15/1674	76/26443	0.001038156
Galactose metabolism	ko00052	25/1674	174/26443	0.001215084
Monobactam biosynthesis	ko00261	8/1674	25/26443	0.00133657
Flavonoid biosynthesis	ko00941	15/1674	84/26443	0.002444545
Linoleic acid metabolism	ko00591	7/1674	26/26443	0.008793403
Stilbenoid, diarylheptanoid and gingerol biosynthesis	ko00945	10/1674	53/26443	0.013306974
Zeatin biosynthesis	ko00908	6/1674	21/26443	0.013521575
Alpha-linolenic acid metabolism	ko00592	13/1674	86/26443	0.022189488
DNA replication	ko03030	15/1674	109/26443	0.026250947
Circadian rhythm - plant	ko04712	12/1674	82/26443	0.036475901

* Pathways with *P*-values ≤0.05 are significantly enriched in DEGs

### Validation and heterologously expression of DEGs

**Fig. 4:**
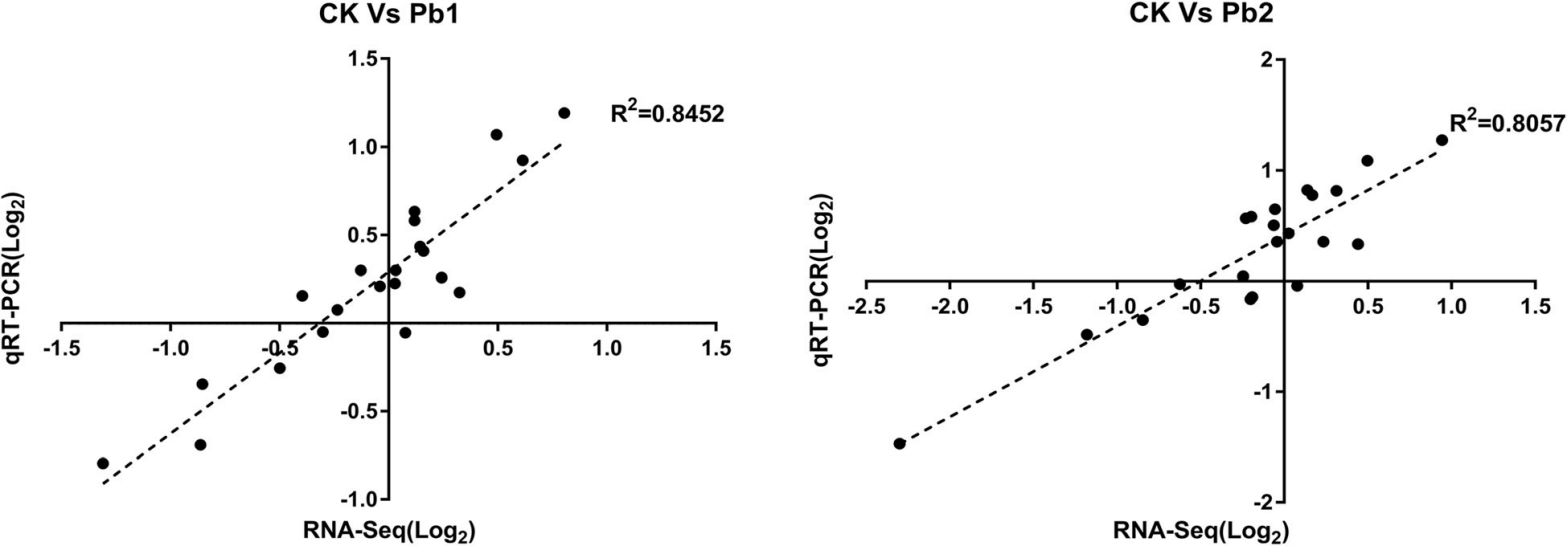
Validation of gene expression ratios between the RNA-Seq and qRT-PCR analyses. Fifteen randomly selected genes were used to examine expression profiles by qRT-PCR using the same RNA employed for RNA-Seq. Each average RNA-Seq expression value was plotted against the corresponding qRT-PCR value and fitted into a linear regression. Both the x- and y-axes are shown on the log2 scale. Each qRT-PCR was performed with three biological replicates

To confirm the accuracy of the RNA-Seq data, 21 unigenes were selected to investigate their transcriptional expression in the leaves via qRT-PCR. The results showed that the RNA-Seq data were well correlated with the qRT-PCR results (Fig. 4), indicating the reliability of the RNA-seq data.

**Fig. 5:**
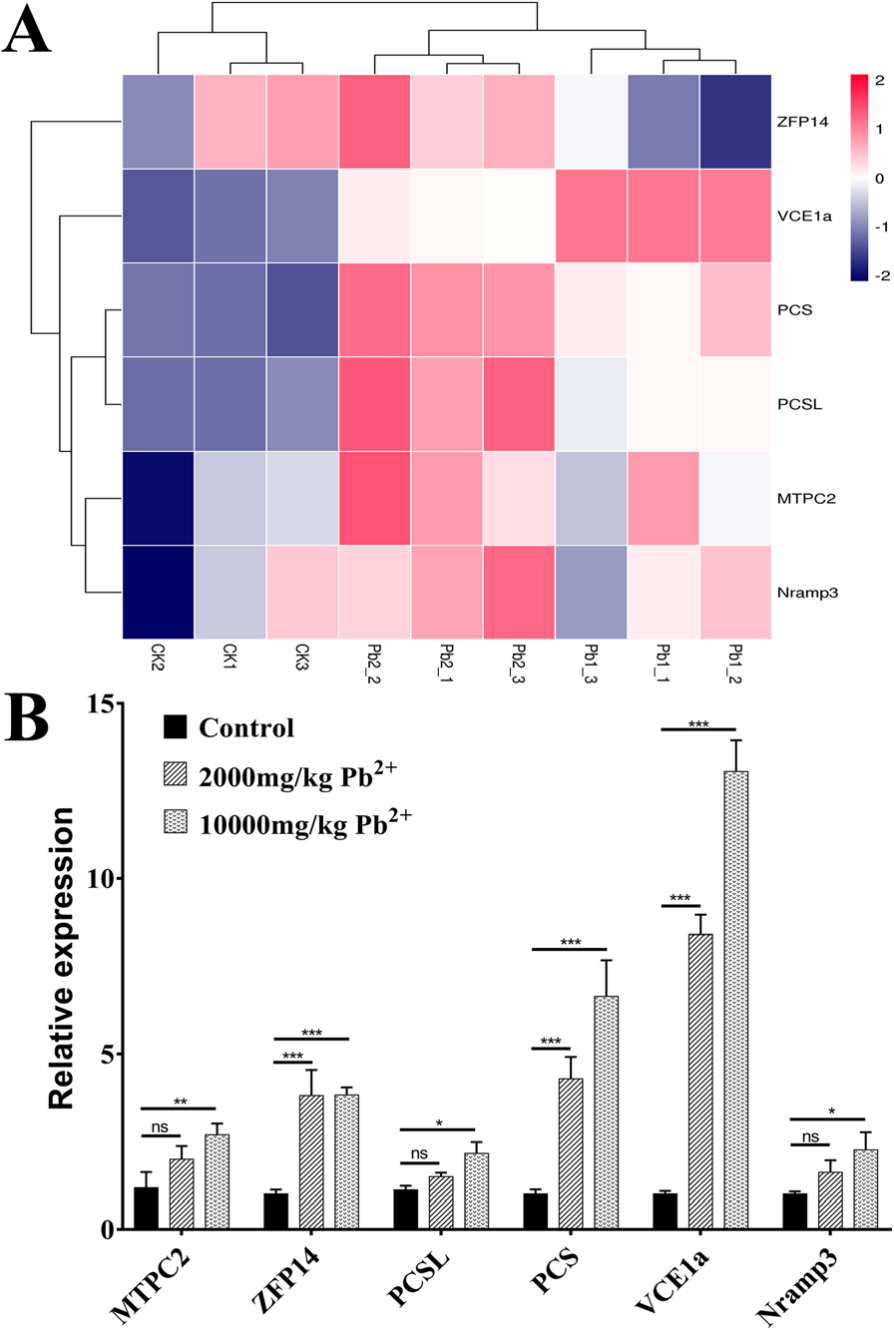
Heat map of the expression levels of 6 DEGs by FPKM (**a**) and by qRT-PCR analysis (**b**). The data shown are the average of three independent experiments. The error bars indicate the standard deviations of the mean. ***, *P* < 0.001; **, *P* < 0.01; *, *P* < 0.05; ns, not significant

Moreover, we have particularly analysed 6 unigenes (*MTPC2*, *ZFP14*, *PCSL*, *VCE1a*, *Nramp3*, and *PCS*) which were regulated in response to Pb stress (Fig. 5a). As results, three upregulated genes (*ZFP14*, *PCS*, and *VCE1a*) were significantly differentially expressed under both assayed concentrations of Pb^2+^. The increase in *ZFP14* expression was the same in the two Pb stress samples, whereas the increases in *PCS* and *VCE1a* expression increased sharply with increasing Pb^2+^ concentrations. In contrast, *MTPC2*, *PCSL* and *Nramp3* were not significantly upregulated in the 2000 mg/kg Pb^2+^ group compared to the control, but the *P*-value for these genes was less than 0.05 in the 10,000 mg/kg Pb^2+^ group (Fig. 5b).

**Fig. 6:**
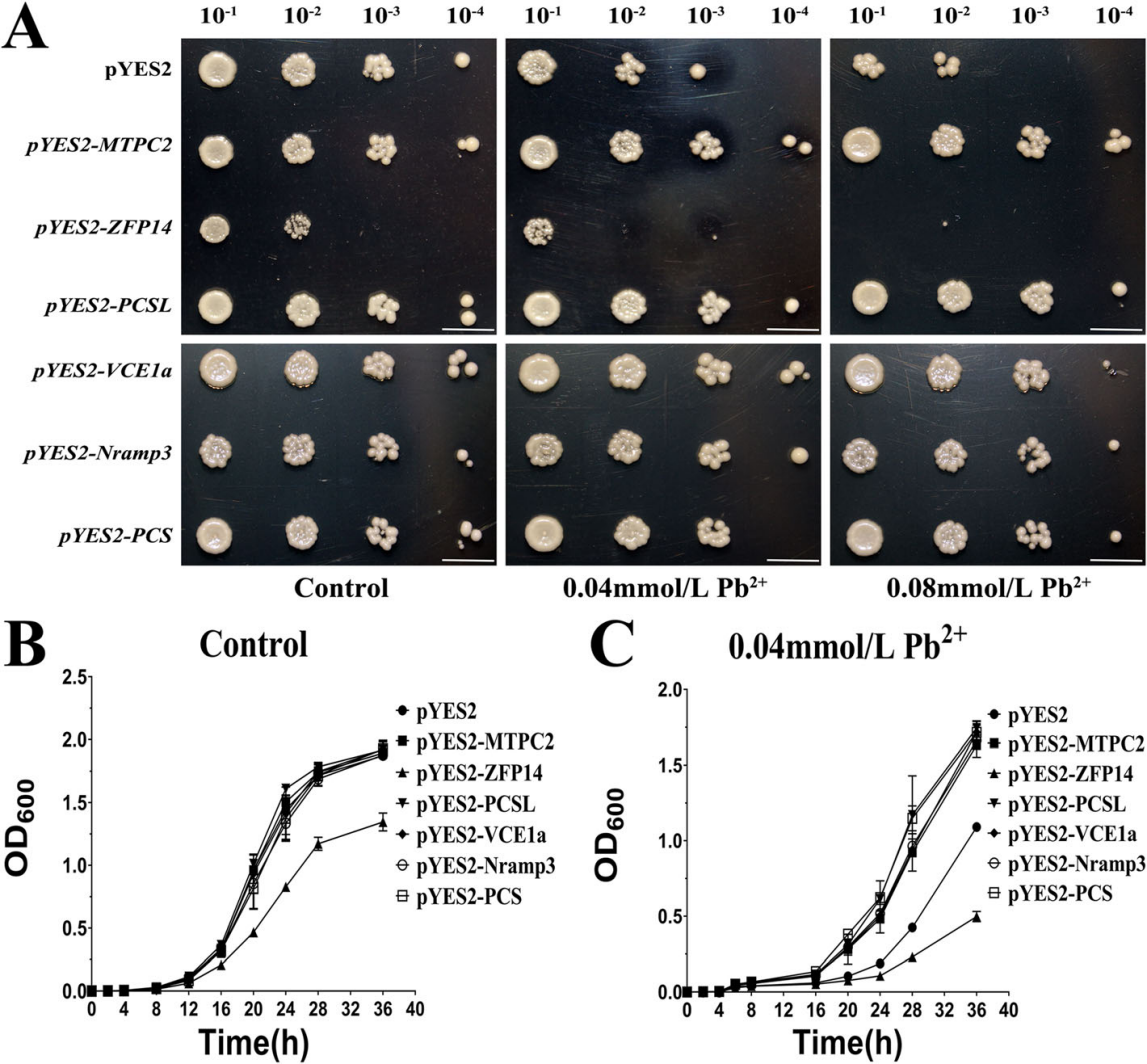
6 Upregulated genes contribute to enhanced tolerance to Pb in yeast. **a** The *Δycf1* strain was transformed with (pYES2), *pYES2-FtMTPC2*, *pYES2-FtZFP14*, *pYES2-FtPCSL*, *pYES2-FtVCE1a*, *pYES2-FtNramp3*, or *pYES2-FtPCS* and grown on SD-Ura (2% galactose) plates with 0, 0.04, or 0.08 mmol/L Pb^2+^ for 4 days. Bar = 1 cm. **b**, **c** Growth curve of strains expressing the 6 upregulated *F. tataricum* genes under 0, 0.04 mmol/L Pb^2+^. The data are presented as the mean ± SD (*n* = 3)

To determine whether these six tartary buckwheat genes were upregulated in response to Pb tolerance, these genes were heterologously expressed in the Pb-sensitive yeast strain *Δycf1*. The results suggested that the expression of *FtMTPC2*, *FtPCSL*, *FtVCE1a*, *FtNramp3* and *FtPCS* strongly increased the Pb tolerance of this strain (Fig. 6). However, compared to the strain harbouring the empty vector (pYES2), Δ*ycf1* cells expressing *FtZFP14* were highly sensitive on medium supplemented with 0.04 and 0.08 mmol/L Pb^2+^ (Fig. 6a), with very little growth on the latter plate. Compared to cells grown on agar-solidified medium, *FtZFP14-*expressing cells cultured in liquid medium supplemented with 0.04 mmol/L Pb^2+^ showed little growth after 48 h (Fig. 6b, c).

## Discussion

Similar to common buckwheat, tartary buckwheat displays high ability to accumulate Pb in contaminated soil [21, 22]. In comparison with previous studies, we first demonstrated that tartary buckwheat could grow in soil with a high concentration of Pb (10,000 mg/kg). Subsequently, we used TEM to show that tartary buckwheat primarily accumulates Pb in the vacuoles of leaves. Although many Pb ions were observed to be absorbed through roots and were present in the cell wall and intercellular spaces, these ions were not stored in root cells and were ultimately transported to leaf vacuoles. Moreover, in order to make clear the mechanism of the tolerance and accumulation of tartary buckwheat leaves under Pb stress, we carried out transcriptome analysis in different concentration of Pb ions. In this study, it generated 88,977 unigenes that comprised 125,203,555 bp and had an average length and N50 of 982.34 bp and 1730 bp, respectively. Our study produced longer sequences and deeper coverage than prior studies with tartary buckwheat [29, 30] (Table 4). Taken together, our present results will not only show a clear physiological understanding of Pb toxicity but provide a valuable database platform for Pb stress research.

**Table 4:** Comparison of our sequencing data with other sequencing data

Database	This research	Chen’s research	Yao’s research
Species	*Fagopyrum tataricum*	*Fagopyrum tataricum*	*Fagopyrum tataricum*
Cultivars of buckwheat	Jiujiang	N/A	Heifeng No. 1 and Xiqiao No. 2
Number of unigenes generated	88,977	45,278	57,800
Total unigene length (bp)	125,203,555	42,818,102	55,189,159
Average length (bp)	982.34	862	954.83
N50 (bp)	1730	1476	1676

As the first barrier of cells, the cell wall protects against Pb entry into the cytoplasm [31]. The cell wall contains polysaccharides and proteins that may serve as binding sites for Pb ions [32]. When metal ions are deposited on the cell wall, their ability to be transported across the membrane into the protoplast is greatly limited, allowing the normal metabolism of plant cells to be maintained [33]. Here, the cellular component group particularly enriched by Pb exposure contained genes encoding cell wall (GO: 0005618) and membrane part (GO: 0044425). In addition, genes related to synthesis of polysaccharides (GO: 0005976, GO: 0044264, GO: 0033692 and GO:0010383) increases in response to Pb stress, causing the cell wall to thicken considerably. Similar observations have been reported for Pb toxicity in *Allium cepa* [34]. We also found that a number of genes associated with changes in organelle membranes and cellular macromolecule metabolic were affected by Pb exposure (Additional file 6: Table S3). Zheng et al. [35] observed that Pb is transported through the apoplastic and symplastic pathways and is detoxified via cell wall sequestration, autophagy and vacuolar compartmentalization as Pb-phosphate. Furthermore, when such binding sites on the cell walls are saturated, excess Pb ions are transferred into the cytoplasm and organelles, i.e., vacuoles and the Golgi apparatus [36, 37]. These organelles show reduced direct contact between Pb ions and enzymes, which prevents enzyme inactivation and blocks biochemical reactions [38]. Accordingly, the cell wall, membrane and organelle work together for the protection of cells under Pb stress.

In response to lead exposure, plants increased the production of ROS by activating different antioxidant enzymes. Overproduction of reactive oxygen species (ROS), which is caused by many environmental stresses (drought, salinity, temperature, flooding and heavy metals), perturbs the structural and functional stability of membrane proteins and disrupts cellular homeostasis [39–41]. The antioxidant system is an important pathway for protecting the cell membrane from injury due to high levels of hydrogen peroxide, superoxide anion and singlet oxygen under Pb stress [42, 43]. In this study, we identified 19 DEGs in tartary buckwheat leaves that were involved in the peroxisome (ko04146) (Additional file 6: Table S3), which is consistent with the increased antioxidant activities reported in previous studies [44, 45]. Venkatachalam et al. [15] found that the accumulation of Pb in plant tissues leads to increases in catalase (CAT), peroxidase (POD) and ascorbate peroxidase (APX) activities. Wang et al. [46] demonstrated that Pb could cause oxidative damage and increase superoxide dismutase (SOD), CAT, POD, glutathione reductase (GR), and APX activities as well as the levels of monodehydroascorbate reductase (MDA) and nonprotein thiols.

Photosynthesis is a highly integrated and regulated process, and the response of photosynthesis to environmental changes is inhibited by heavy metals such as Pb [47, 48]. Chen et al. [49] reported that in rice, the most basic and apparent symptoms of Pb-induced toxicity include leaf chlorosis; stunting; reduced net photosynthesis, stomatal conductance, and leaf transpiration; and less accumulation of photosynthetic pigments such as chlorophyll (Chl) a, Chl b and carotenoids. Furthermore, by maintaining the balance of photosynthetic energy, increases in chlorophyll content and the number of chloroplasts may enhance plant tolerance to Pb [16, 26]. In our study, the ‘photosynthesis’ (ko00195) KEGG pathway was enriched (Additional file 6: Table S3), and a number of DEGs common to both the CK vs Pb1 and CK vs Pb2 comparisons were involved in the photosystem (PS) II, PS I and F-type ATPase pathways. The major effects of Pb stress in plants involve potential damage to the oxygen-evolving complex (OEC) and the inhibition of PS I and II activity [50]. In accordance with this notion, five genes involved in the PS I and PS II pathways were downregulated with different fold changes based on our transcriptome results. Similarly, almost all of these DEGs, including the PSII core proteins D1, D2, CP43 and CP47, which are considered to have the closest relationship with oxygen production, were downregulated [51]. Therefore, impaired photosynthesis is a way to reduce oxygen production and protect against oxidative damage by reducing the related genes in chloroplasts. Taken together, these results suggest that the regulation of the photosynthesis pathway may be a common response to Pb stress among various plant species.

Together with the wide range of adaptive strategies induced by Pb stress, the plants are able to acquire other detoxification and defence mechanisms through gene regulation. On one hand, plant possesses a nonenzymatic antioxidative mechanism of defence against Pb exposure [52]. Our result showed that the phenylpropanoid biosynthesis (ko00940) was one of the most significantly enriched with DEGs in the tartary buckwheat leaves under Pb stress (Table 3 and S4). In this pathway, phenylalanine ammonium lyase (PAL) is the key regulatory enzyme in altering the biosynthesis and accumulation of flavonoids and lignin [53]. Moreover, the branched biochemical reactions of phenylpropanoid biosynthesis, including flavonoid biosynthesis (ko00941) and carotenoid biosynthesis (ko00906), providing a number of important phenolic compounds (anthocyanin, carotenoids and flavonoids) [54]. These metabolites protect the plant against Pb-induced oxidative stress by scavenging H_2_O_2_ and active free radicals [55]. On the other hand, the initial perception of plants to heavy metal can trigger signal transduction, and initiate gene expression and cellular processes involved in acclimation to stress. Environmental stressors are transmitted through hormone signalling and MAP kinase (MAPK) pathways to target transcription factors (TFs) [56]. Plant hormones are signalling molecules in plants exposed to various tolerances, including Pb stress. The hormones related to heavy metals include abscisic acid (ABA), auxin, jasmonic acid (JA), and salicylic acid (SA) [57]. Under HMs stress, the plant hormone concentration was elevated, thereby upregulating the expression of MAPKs and GSH-metabolic genes and stimulating GSH biosynthesis, which is involved in signalling pathways and stress responses [58]. In our study, a majority of DEGs were enriched in the pathways involved in plant hormone signal transduction, MAPK signalling and glutathione metabolism (Table S4). Therefore, signalling from upstream second messengers and hormones is transduced to downstream acceptors (MAPKs). The MAPK signalling pathways play a pivotal role in this process and interact with other signalling molecules to mediate crosstalk among plant signalling systems to facilitate adaptation and coordinate plant responses to various stressors [59].

Moreover, some DEGs that were not significantly enriched according to GO or KEGG analyses may nonetheless be involved in regulating Pb tolerance and hyperaccumulation under Pb stress. Phytochelatins (PCs), which are synthesized from glutathione (GSH) by phytochelatin synthetase (PCS) [60], form mercaptide bonds with various metals and are ultimately transported into the vacuolar space [61, 62]. To the results of previous studies, natural resistance-associated macrophage proteins (Nramps) [63, 64], metal tolerance protein (MTP) [65, 66] and vacuole transporter (YCF) [67] are all membrane transporters that have been proved to enhance the tolerance or hyperaccumulation in plant. To verify whether these genes participate in the same process in tartary buckwheat, these genes were heterologously expressed in yeast, and the results were consistent with the above mechanism (Fig. 6).

**Fig. 7:**
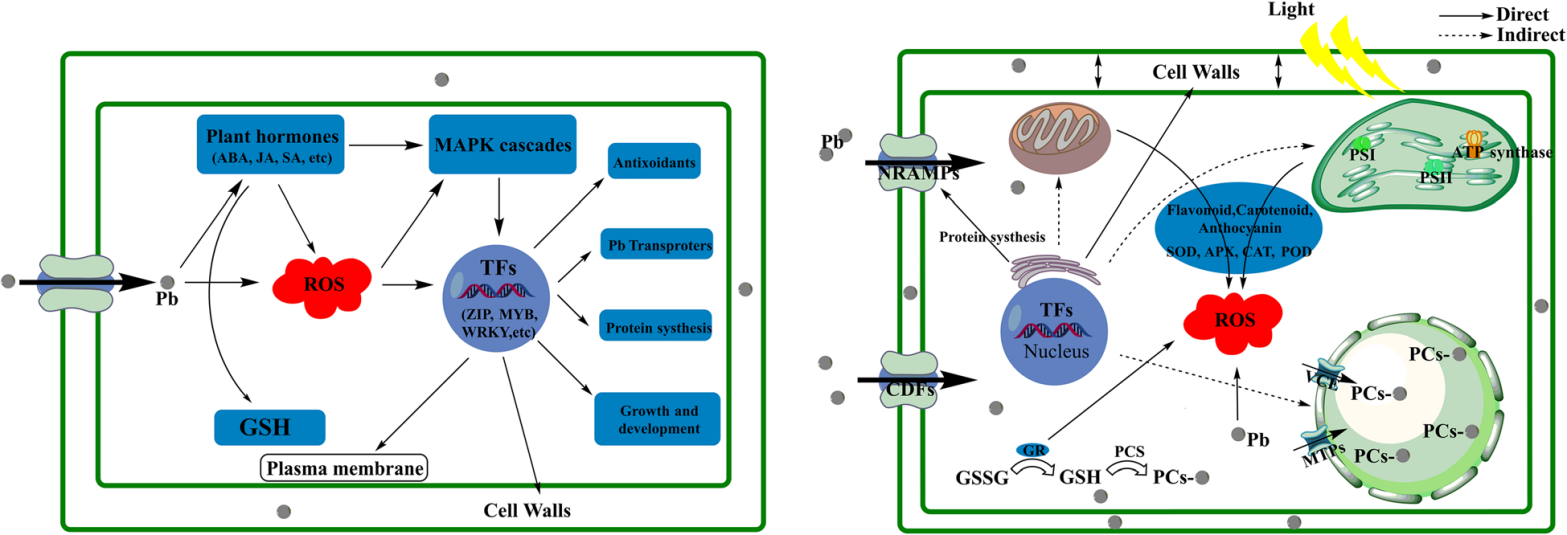
Schematic diagram of the response to Pb^2+^ in the leaves of *F. tataricum*

Based on the present results, we inferred that a variety of metabolic processes in tartary buckwheat leaves are affected during Pb stress. As illustrated in Fig. 7, after Pb ions are transported to the leaves from the roots, a portion of Pb ions bind to cell walls. Moreover, the vast majority of these ions are transported into the cytoplasmic matrix by cation diffusion facilitators (CDFs), Nramps and other ion transporters through iron or water channels [68]. Once Pb ions enter plant cells, the concentrations of plant hormones change, which results in the accumulation of GSH. ROS interact with plant hormones and participate in MAPK cascades to activate TFs. Consequently, the genes that changed in response to Pb stress were regulated by TFs to defend against and mitigate Pb exposure. Subsequently, cells maintain the cellular homeostasis by antioxidative enzymes, including APX, CAT, SOD, POD, and GR, in all types of organelles. Then, Pb ions are chelated by GSH, PCs and other compounds and are then transported into vacuoles by VCE (a homologue of YCF), MTPs and related transporters. These findings suggest the metabolic process involved in the Pb response of tartary buckwheat leaves and the transport and accumulation of Pb ions.

## Conclusions

In summary, the results of our study show that tartary buckwheat leaves are the primary storage tissue for Pb and provide transcriptome data for tartary buckwheat leaves in response to Pb stress. Furthermore, we identified 374 DEGs that were significantly associated with the primary events of Pb exposure and the response to Pb treatment during the early growth stage of tartary buckwheat leaves. According to GO and KEGG pathway enrichment analyses, these DEGs are primarily associated with primary defence mechanisms, such as cell walls, plant hormone signal transduction, the antioxidant system and photosynthesis. In addition, these DEGs are involved in hyperaccumulation mechanisms, e.g., TFs, metal iron binding and membrane transport proteins, as demonstrated by their heterologous expression in yeast. This study specifically explored the fundamental Pb tolerance and hyperaccumulation mechanisms in the leaves of tartary buckwheat, providing new information for further research on the molecular mechanisms of the response to Pb stress in tartary buckwheat.

## Methods

### Plant materials and growth conditions

The *Fagopyrum tataricum* ‘Jiujiang’ cultivar used in the present study was acquired from the Yan Chai Lab of Northwest A&F University. The seed growth conditions were similar to those described by Tamura [21], with some changes. Seeds were prevernalized at 4 °C for one week, soaked in deionized water for 2 h at 45 °C, and sown into 40 g PEAT substrate (PINDSTRUP, Latvia; https://www.pindstrup.com/) without any heavy metal pollution, with four seeds per pot (30 cm diameter). The plants were grown in a greenhouse at 60% relative humidity with 12 h of light and a 25/15 °C artificial day/night air temperature. After culture for 15 days, the cotyledons of buckwheat had completely unfolded, and the plants were treated with 0, 1000, 2000, 5000, or 10,000 mg/kg Pb (NO_3_)_2_ depending on the dry weight of the substrate soil. Each group was repeated ten times. According to the previous study [21], we chose and collected leaf tissue samples 72 h after treatment with 0, 2000, or 10,000 mg/kg Pb (NO_3_)_2_ for Illumina deep sequencing (three biological replicates) and qRT-PCR validation (three biological replicates). We defined the 0 mg/kg Pb (NO_3_)_2_ treatment as the control ‘CK’, the 2000 mg/kg Pb (NO_3_)_2_ treatment as ‘Pb1’, and the 10,000 mg/kg Pb (NO_3_)_2_ treatment as ‘Pb2’. The samples were immediately frozen in liquid nitrogen and stored at − 80 °C until use. The remaining plants were continuously cultivated and harvested before anthesis to assess the accumulation of Pb in plant materials. The plant materials were washed with deionized water and dried for 48 h at 80 °C, after which they were weighed, ground in a mill, and then digested with concentrated HNO_3_:HClO_4_ (4:1) in a microwave oven. After digestion, the solution was diluted to 25 mL with 1% HNO_3_ and filtered. Subsequently, the samples were analysed using a Hitachi Z-2000 atomic absorption spectrometer (Hitachi, Japan).

### Ultrastructural localization of Pb using TEM

This part references Małgorzata’s method [69] with some changes. One- to two-millimetre sections of the control and 10,000 mg/kg Pb (NO_3_)_2_-treated leaves, stems, and roots were fixed in 4% glutaraldehyde (v/v) in 0.2 mol/L sodium phosphate buffer (49 mL 0.2 mol/L Na_2_HPO_4_•12H_2_O with 51 mL NaH_2_PO_4_•2H_2_O in a total of 100 mL) at pH 6.8 at 4 °C for 12 h. The tissues were rinsed with 0.1 mol/L phosphate buffer (pH 6.8) six times (10 min each time) at room temperature, postfixed in 1% (v/w) OsO_4_ at 4 °C for 2 h and then rinsed six times (10 min each time) in 0.1 mol/L phosphate buffer solution (pH 6.8). The samples were dehydrated using graded acetone and ethanol series (30, 50, 70, 80 and 90%) at room temperature, infiltrated, embedded in LR white resin and cut into ultrathin sections (~ 90 nm) using a Leica EM UC7 microtome (Leica, Nussloch, Germany). The sections were collected on copper grids, dusted with coal, and finally observed using a JEOL JEM-1230 transmission electron microscope (JEOL, Tokyo, Japan) at an accelerating voltage of 80 keV.

### RNA extraction, cDNA library preparation and sequencing

Total RNA was extracted from frozen leaf tissue using the Quick RNA isolation kit (Huayueyang, Beijing, China), which uses RNase-free DNase I to remove residual genomic DNA. The RNA quantities and qualities were assessed using an Agilent Bioanalyzer 2100 (Agilent Technologies, Santa Clara, CA, USA), and the RIN values were greater than 7. RNA integrity was assessed by agarose gel electrophoresis (1% gel with the addition of SYBR Green dye). To obtain a global and high-quality overview of the tartary buckwheat transcriptome, individual RNAs from leaf tissues from three biological replicate plants exposed to three Pb concentrations were used for library construction.

cDNA libraries were constructed using a TruSeq™ RNA Sample Prep kit (Illumina, USA) following the manufacturer’s instructions. Briefly, mRNA was purified from total RNA using Dynabeads oligo (dT)25 (Life Technologies, USA). Subsequently, poly(A) mRNA was purified from 25 μg of pooled total RNA using oligo (dT) magnetic beads and was cut into short fragments of approximately 300 bp in fragmentation buffer. The resulting mRNA was fragmented and reverse transcribed into first-strand cDNA using random hexamer primers, after which second-strand cDNA was synthesized. The double-stranded cDNA was purified and ligated to adaptors for Illumina paired-end sequencing. After agarose gel electrophoresis, suitable fragments (> 200 bp) were chosen for PCR amplification to create the final cDNA libraries. The resulting cDNA libraries were sequenced at Shanghai Majorbio Biopharm Technology Co., Ltd. (Shanghai, China) using an Illumina HiSeq™ 4000 sequencing system.

### Data filtering, de novo assembly and annotation

The raw image data from Illumina sequencing were transformed into raw reads by base calling. The raw reads were filtered to obtain high-quality clean data and assembled using the program Trinity (Version: V2.5.0) [70] to generate contigs. Redundant sequences were removed, and the resulting contigs were connected into scaffolds to obtain unigenes. The raw data were deposited in the National Center for Biotechnology Information (NCBI) Sequence Read Archive (SRA) (Accession Number: PRJNA515389). Before functional annotation, the assembled transcripts (unigenes) were analysed using Trinity (http://trinityrnaseq.sourceforge.net/analysis/extract_proteins_from_trinity_transcripts.html) to predict open reading frames (ORFs). All nine libraries were assembled using the Trinity method due to the low quality of the reference genome database for tartary buckwheat. The functional annotations of all the unigenes were compared with sequences in the NR protein database (http://www.ncbi.nlm.nih.gov/), Clusters of Orthologous Groups (COG) protein database (http://www.ncbi.nlm.nih.gov/COG/), and KEGG database (http://www.genome.jp/kegg/) using BLASTx (E-value) [71]. The Blast2GO program (Version: 2.5.0) was used to analyse GO annotations for the unigenes. Functional annotations were performed by aligning the unigenes to those present in the NCBI nonredundant (NR), COG, GO, String, Swiss-Prot and KEGG databases, with an E-value of <1e-5 (Additional file 7: Table S4). The number of unigenes associated with each GO term was then calculated for the BP, MF, and CC categories. The unigene sequences were also aligned to the COG database to predict and classify potential functions. In addition, the COG and KEGG databases were used to complement the GO functional characterizations and determine the sequence directions of the unigenes.

### DEGs and enrichment analysis

All clean sequence reads were mapped back to the transcriptome assembly using Bowtie2 (Version: 2.3.4) [72], and the read counts were then normalized as transcripts per million reads (FPKM) to calculate the unigene expression level by RSEM (http://deweylab.biostat.wisc.edu/rsem/) (Version: 1.2.31). The DEGs among the three samples were analysed via gene read counts using edgeR (http://www.bioconductor.org/packages/2.12/bioc/html/edgeR.html) (Version: 3.14.0) under the following standard parameters: FDR < 0.05 and log2|FC| > = 1. GO and KEGG pathway enrichment analyses were conducted via hypergeometric distribution testing using GOATOOLS tools (Version: 0.6.5). BH (fdr correction with Benjamini/Hochberg) correction was used to adjust *P*-values. Functional clusters were considered significantly enriched when the corrected P-value was smaller than 0.05.

### qRT-PCR validation and expression analysis

Twenty-one genes, including 15 DEGs, were randomly selected, and 6 Pb-regulated genes were used for further validation by qRT-PCR. Total RNA extraction and genomic DNA removal were performed as described above. First-strand cDNA was synthesized from 1 μg of RNA using a HiScript® 1st Strand cDNA Synthesis kit (Vazyme, China). The cDNA products were then diluted tenfold with nuclease-free water for use as a template for qRT-PCR, which was performed using the ChamQ™ SYBR Green Master mix (Vazyme, China) with a CFX96 Real-Time PCR Detection System (Bio-Rad). The specific primer pairs used for qRT-PCR are listed in Additional file 8: Table S5. For qRT-PCR validation, the primer specificity was tested by PCR (Additional file 9: Figure S4). All samples were normalized to the *CACS* gene [73]. The DEG expression fold change was calculated based on the threshold cycle (Ct), where ΔCt = Ct_target_ − Ct_CACS_ and Δ (ΔCt) = ΔCt_Control_ − ΔCt_Indicated condition_.

### Pb resistance in yeast cells

The coding sequences of *FtMTPC2*, *FtZFP14*, *FtPCSL*, *FtVCE1a*, *FtNramp3*, and *FtPCS1* from the cDNA library constructed by RNA-Seq were amplified, and the fragments were subcloned into pEASY-Blunt Zero (TransGen Biotech, China) and sequenced. The correct coding DNA sequence (CDS) fragments were cloned into a pYES2 vector using the ClonExpress II One Step Cloning kit (Vazyme, China) for expression in yeast. Cells of the Pb- and cadmium-sensitive yeast strain *Δycf1* (*MATa his3Δ1 leu2Δ0 met15Δ0 ura3Δ0 YCF1*::*kanMX4*) carrying the empty vector (pYES2), *pYES2-FtMTPC2*, *pYES2-FtZFP14*, *pYES2-FtPCSL*, *pYES2-FtVCE1a*, *pYES2-FtNramp3*, or *pYES2-FtPCS* were grown in medium at 30 °C. To analyse Pb resistance, the yeast cells were cultured in SD-Ura (2% glucose) medium at 30 °C until reaching an OD_600_ of 2.0 and then serially diluted (10^− 1^, 10^− 2^, 10^− 3^, 10^− 4^) with 1× TE buffer. The cells were spotted onto an SD-Ura (2% galactose) medium plate with Pb^2+^ (0, 40, or 80 μmol/L) and cultured at 30 °C in an incubator for 4 d. Yeast cells were inoculated onto SD-Ura (2% galactose) medium with 0 or 40 μmol/L Pb^2+^ at a 1:1000 ratio to determine the growth curve.

### Data analysis

All values are expressed as the mean ± standard deviation from three individual experiments. The data analysis was performed using one-way analysis of variance (ANOVA) followed by Tukey’s test with GraphPad Prism 6. Differences were considered significant at *P* < 0.05.

## Supplementary information

**Additional file 1: Figure S1.** Physiological aspects of tartary buckwheat leaves under Pb stress at different concentrations.

**Additional file 2: Figure S2.** Sequence length distribution of the unigenes.

**Additional file 3: Table S1.** Annotations of all unigenes.

**Additional file 4: Figure S3.** Species and similarity distribution based on the best hit.

**Additional file 5: Table S2.** Common regulated genes in the CK vs Pb1 and CK vs Pb2 comparisons.

**Additional file 6: Table S3.** GO enrichment analysis of DEGs.

**Additional file 7: Table S4.** KEGG pathway enrichment analysis of DEGs.

**Additional file 8: Table S5.** Primers used for quantitative real-time PCR analysis.

**Additional file 9: Figure S4.** The primer specificity test for qPCR validation.

## Data Availability

All data generated or analysed during this study are included in the manuscript and its Additional files. The sequencing dataset used in the study is available in the Sequence Read Archive of the NCBI database under BioProject: PRJNA515389 (https://www.ncbi.nlm.nih.gov/bioproject/PRJNA515389/), which will be made public after publication.

## Abbreviations

AAS: Atomic absorption spectrophotometry
Al: Aluminium
APX: Ascorbate peroxidase
BP: Biological process
BZIP: Basic region-leucine zipper
CAT: Catalase
CC: Cellular component
DEGs: Differentially expressed genes
DMT: Divalent metal transporter
ERF: Ethylene-responsive factor
FPKM: Reads per kilobase of exon per million reads mapped
GARP: Glutamic acid-rich protein
GO: Gene Ontology
GR: Glutathione reductase
GSH: Glutathione
KEGG: Kyoto Encyclopedia of Genes and Genomes
MDA: Monodehydroascorbate reductase
MF: Molecular function
MTPC2: Metal transporter protein C2
NGS: Next-generation sequencing
NR: Nonredundant protein database
Nramp3: Natural resistance-associated macrophage protein 3
OEC: Oxygen-evolving complex
ORFs: Open reading frames
Pb: Lead
PCS: Phytochelatin synthetase
PCs: Phytochelatins
PCSL: Phytochelatin synthetase-like family protein
POD: Peroxidase
PS: Photosystem
qRT-PCR: Quantitative real-time PCR
RNA-Seq: RNA deep sequencing
ROS: Reactive oxygen species
RSEM: RNA-Seq by Expectation-Maximization
SOD: Superoxide dismutase
TEM: Transmission electron microscope
VCE1a: Vacuolar cation/proton exchanger 1a
YCF1: Hypothetical chloroplast open reading frame 1
ZFP14: CCCH-type zinc finger protein 14
