# Supplementary figures and images for: Transcriptome profiling of Fagopyrum tataricum leaves in response to lead stress
Source: BMC Plant Biol. 2020 Feb 3;20:54. doi: 10.1186/s12870-020-2265-1 (PMC6998078; doi:10.1186/s12870-020-2265-1)

## Slide 1
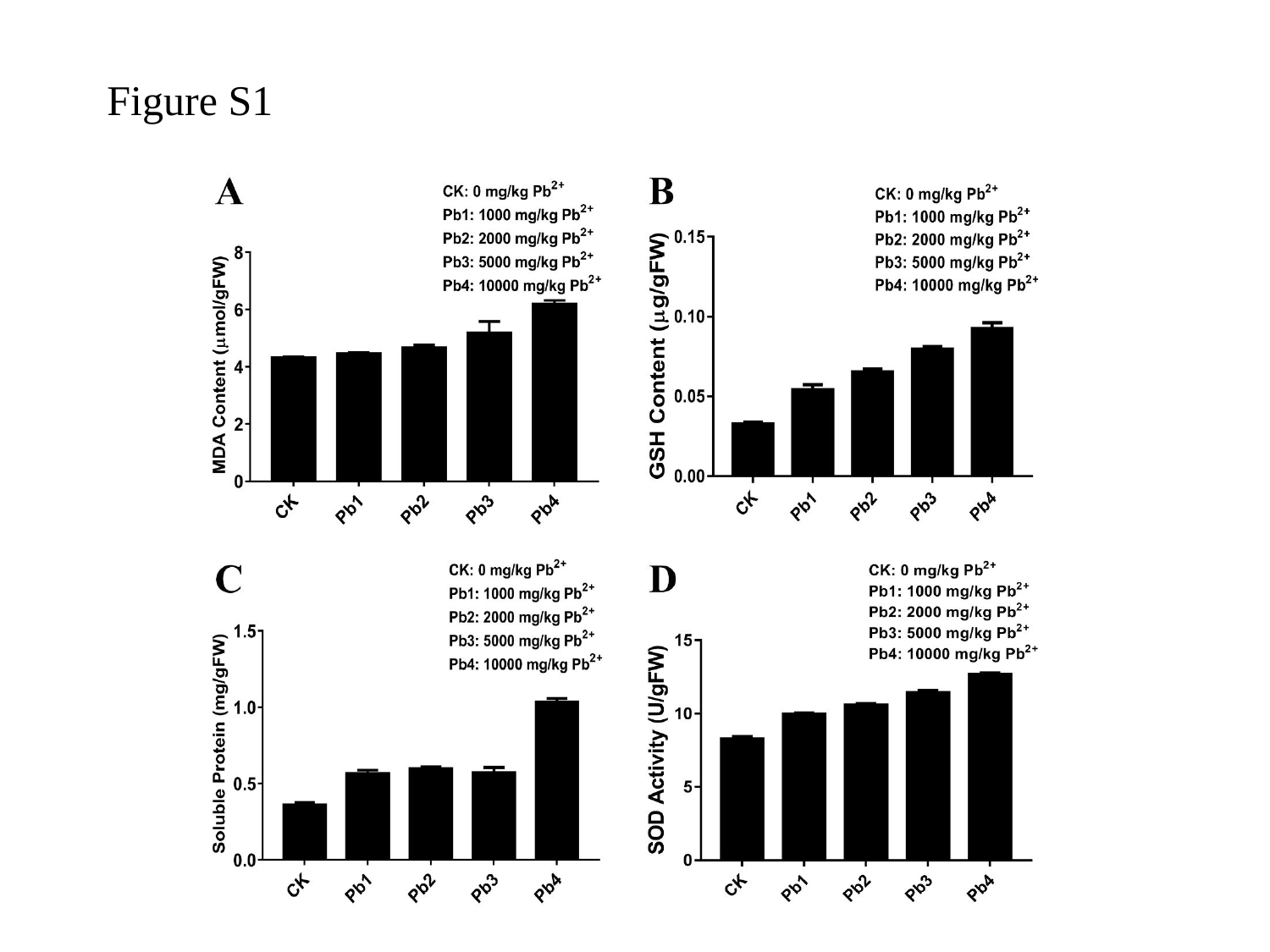

Figure S1

Supplement: Supplementary file 1 — Additional file 1: Figure S1. Physiological aspects of tartary buckwheat leaves under Pb stress at different concentrations. [file 12870_2020_2265_MOESM1_ESM.pptx]

## Slide 1
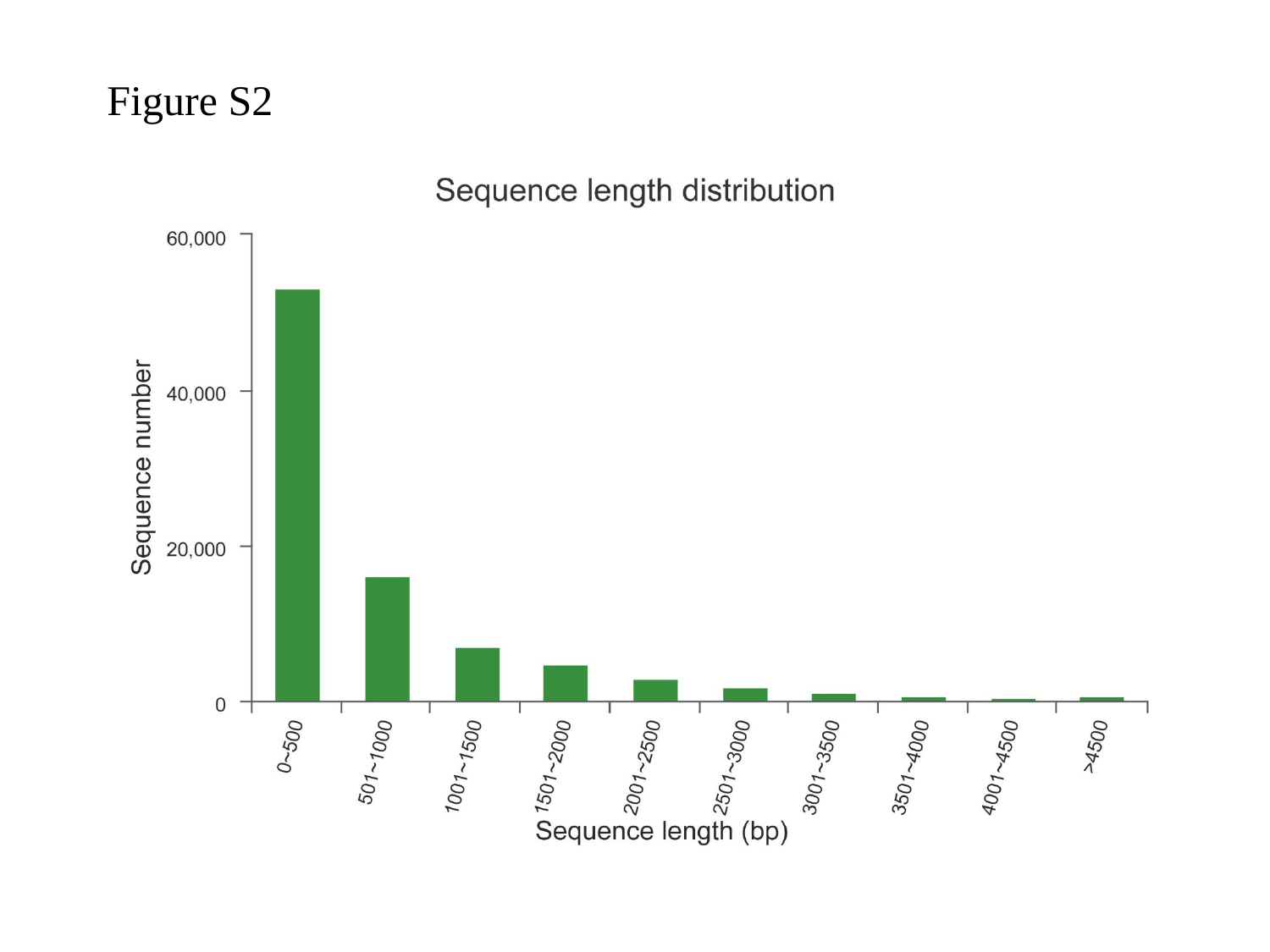

Figure S2

Supplement: Supplementary file 2 — Additional file 2: Figure S2. Sequence length distribution of the unigenes. [file 12870_2020_2265_MOESM2_ESM.pptx]

## Slide 1
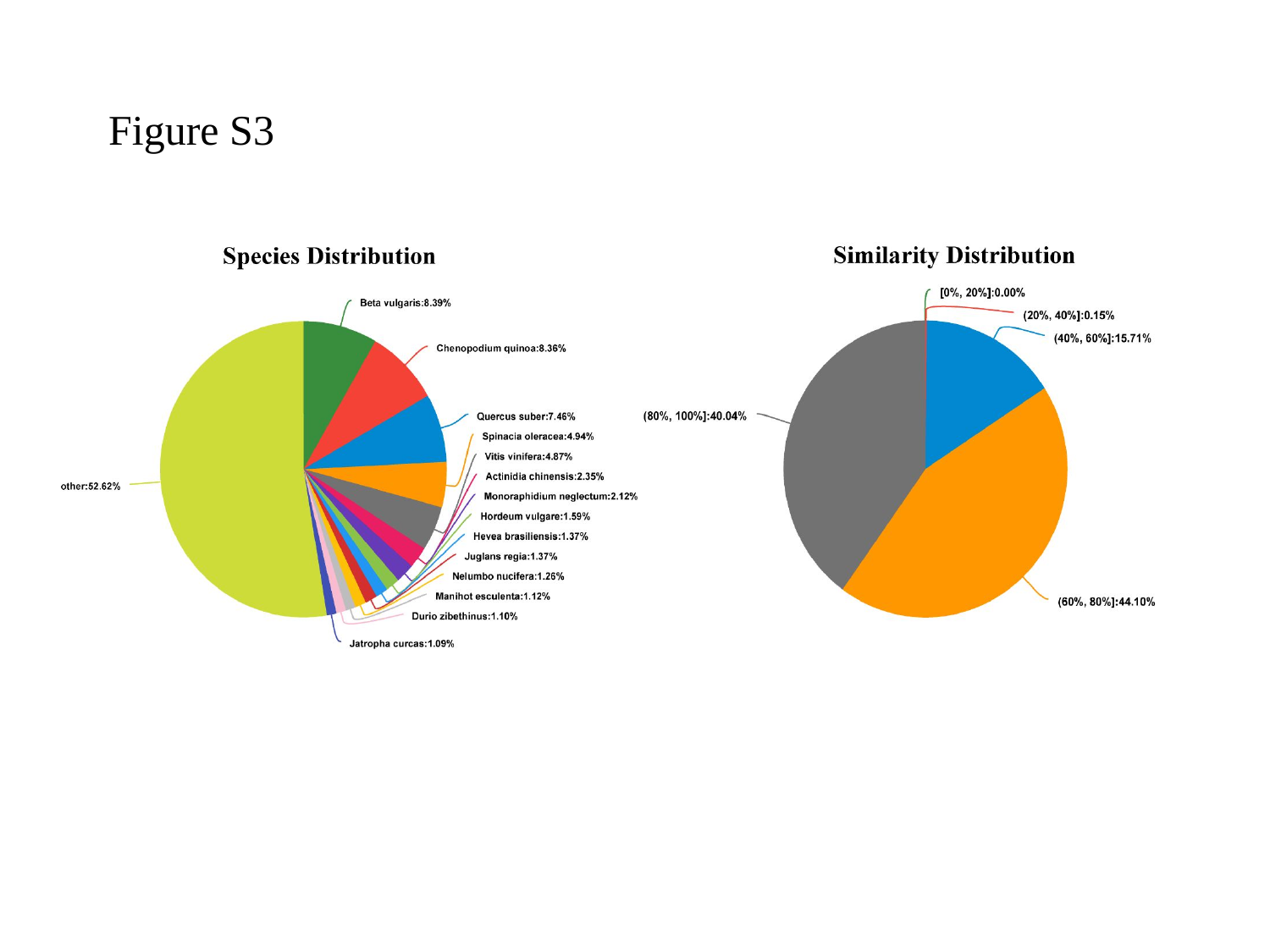

Figure S3

Supplement: Supplementary file 4 — Additional file 4: Figure S3. Species and similarity distribution based on the best hit. [file 12870_2020_2265_MOESM4_ESM.pptx]

## Slide 1
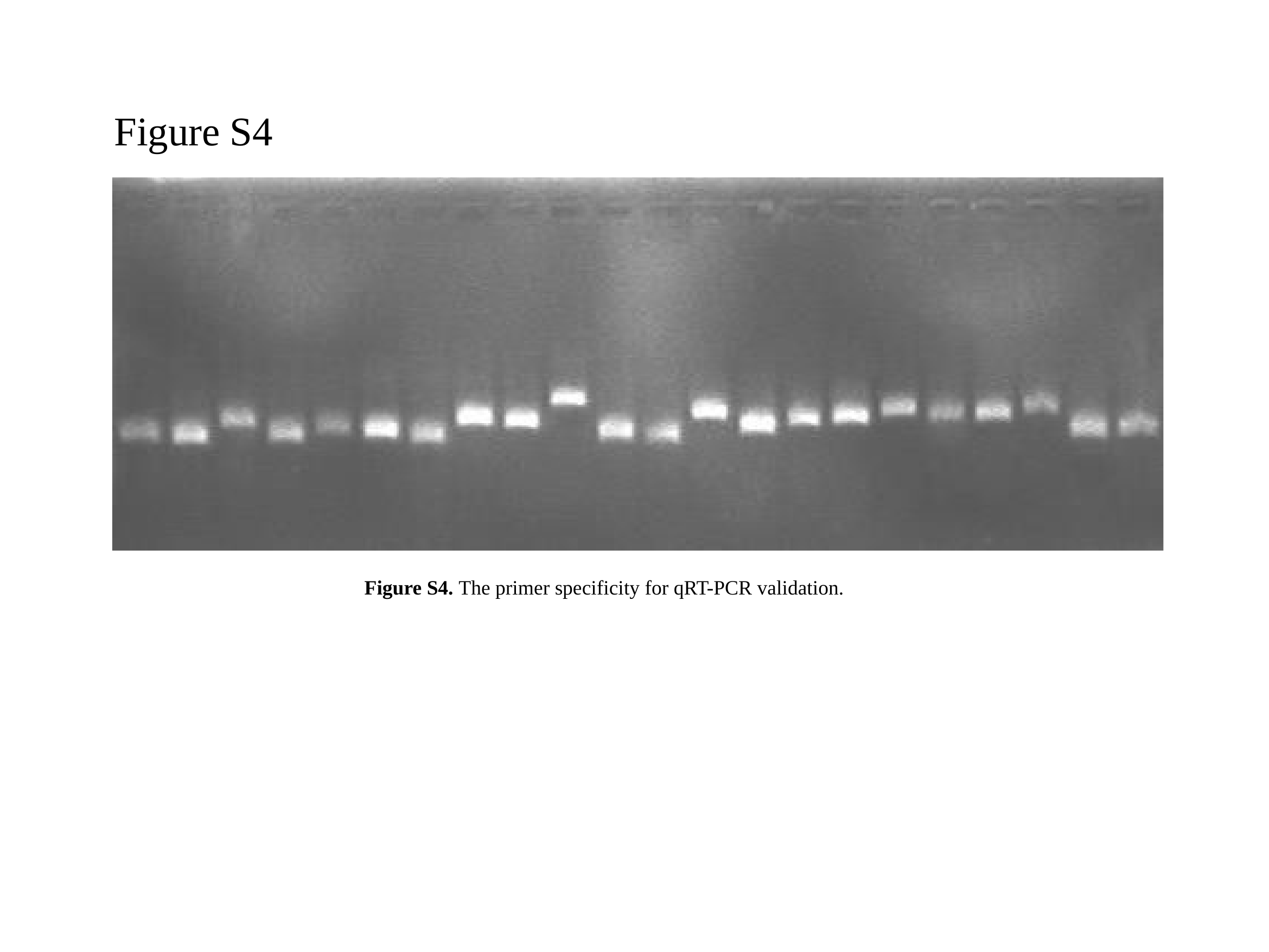

Figure S4
Figure S4. The primer specificity for qRT-PCR validation.

Supplement: Supplementary file 9 — Additional file 9: Figure S4. The primer specificity test for qPCR validation. [file 12870_2020_2265_MOESM9_ESM.pptx]
